# Size effects in the magnetic anisotropy of embedded cobalt nanoparticles: from shape to surface

**DOI:** 10.1038/srep14749

**Published:** 2015-10-06

**Authors:** Simón Oyarzún, Alexandre Tamion, Florent Tournus, Véronique Dupuis, Matthias Hillenkamp

**Affiliations:** 1 Institut Lumière Matière, UMR5306 Université Lyon 1-CNRS, Université de Lyon, 69622 Villeurbanne cedex, France

## Abstract

Strong size-dependent variations of the magnetic anisotropy of embedded cobalt clusters are evidenced quantitatively by combining magnetic experiments and advanced data treatment. The obtained values are discussed in the frame of two theoretical models that demonstrate the decisive role of the shape in larger nanoparticles and the predominant role of the surface anisotropy in clusters below 3 nm diameter.

Size effects, i.e. size dependent variations of the physical and chemical properties, are at the very heart of nanoscience^1^. This is all the more true for magnetic nanoparticles with the impact of miniaturization in magnetic data treatment and storage devices or the increasing importance of magnetic nanoparticles in biomedical applications^2^. Stern-Gerlach experiments today considered seminal have demonstrated size dependent variations of the magnetic moment in transition metal clusters^3^, whereas the possibility to investigate trapped mass-selected clusters with synchrotron radiation and to thus study the partitioning of magnetic moment into spin and orbital components has renewed the interest in gas-phase clusters^4^,^5^.

Present and future applications, however, demand supporting or embedding the magnetic nanoparticles. For such systems a large number of experiments have significantly advanced our knowledge but the complexity of the possible variations of both geometric and electronic structure of the cluster due to the interactions with the support or the matrix are responsible for the large number of open questions and contradictions. Especially the magnetic anisotropy energy (MAE) is very sensitive on the cluster size as well as on geometric distortions and alloying or electronic hybridization at the interface^6^,^7^,^8^,^9^. The MAE is the energy needed to reverse the nanoparticle magnetization and is commonly defined as 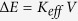 with the effective anisotropy constant *K*_*eff*_ and the volume *V*. Note that *K*_*eff*_ is called anisotropy constant for historical reasons. The term anisotropy coefficient may seem more adequate given its size and morphology dependence. Reducing the size of the nanoparticle can drastically increase *K*_*eff*_, often modeled through a surface term proportional to the inverse diameter^10^. The detailed size-dependent variations of the different contributions to the magnetic anisotropy are, however, still unclear today.

Theoretical investigations on the other hand allow a deeper understanding of the nanoscale origins of magnetic anisotropy which is an intricate balance of relatively large components of opposite sign from different layers^11^,^12^,^13^. Notably the reduced coordination of surface atoms produces complex redistributions of the spin-polarized electronic density and thus changes the spin-orbit energy, responsible for the magnetic anisotropy^14^. Additional contributions to the MAE may be due to the particle shape or to proximity effects at the interface^15^.

There are today uncertainties over orders of magnitude in the magnetic anisotropy energies of cobalt nanoparticles^12^,^16^,^17^,^18^,^19^,^20^,^21^, derived from both experiment and theory, that hinder a comprehensive understanding of size effects and underline the necessity for reliable experimental data. Values as low as 0.1 kJ/m^3^^21^ and as high as 3000 kJ/m^3^^16^ can be found in the literature. In this article we show how the detailed data treatment of magnetic experiments performed on well-defined cluster-assembled nanostructures reveal size effects in the MAE in cobalt nanoparticles embedded in copper. We apply experimental routines previously developed^22^,^23^,^24^ for the first time to obtain reliable ensemble data on the size-varying magnetic anisotropy of embedded cobalt nanoparticles. The unambiguous, consistent and redundant determination of all magnetic values in question and their good agreement with geometric parameters underline the high quality of our data.

## Experimental

**Figure 1:**
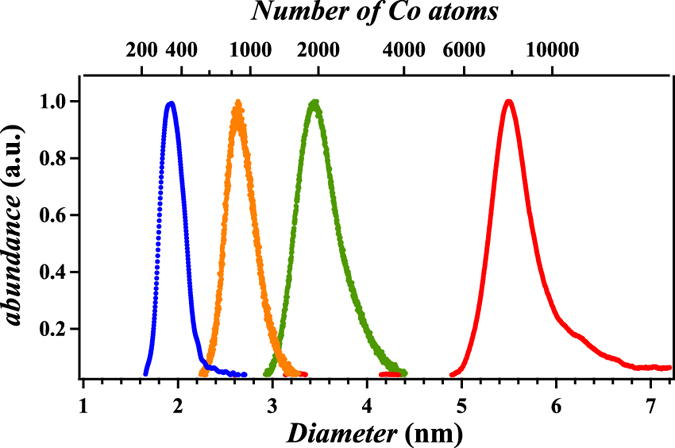
TOF-MS spectra of cobalt clusters at different mean sizes (1.9 nm–5.5 nm) as produced by the magnetron cluster source.

The samples consist of cobalt nanoparticles embedded in copper matrices, where the mean particle diameter was varied from 1.9 nm to 5.5 nm (cf. Fig. 1). The samples were prepared according to the strategy of cluster-assembled materials. The experimental setup with typical operating conditions is described in detail elsewhere^25^ and is only sketched briefly. Cobalt cluster ions are generated in the gas phase using a home-built magnetron cluster source (based on the principle introduced by Haberland *et al.*^26^) and guided towards the deposition chamber housing the silicon substrate surface with its native oxide layer for sample preparation. Time-of-flight mass spectrometry (TOF-MS) is used for *in-situ* characterization of the cluster ions in the gas phase and shows no trace of cluster oxidation or other complexes apart form *CoAr*^*+*^ for sizes up to *Co*_150_. The kinetic energy of the clusters is adjusted below 0.5 eV/atom in order to ensure soft-landing conditions without fragmentation^27^. The copper matrix is evaporated in a commercial electron beam evaporator and co-deposited simultaneously at room temperature with the clusters to reach thicknesses around typically 200 nm. The cobalt concentration can be adjusted by varying the matrix deposition rate and is reduced to below 0.5 at.% to avoid magnetic interactions^28^. No further heat treatment was performed on the samples discussed here. Magnetic measurements were performed in a SQUID MPMS-XL5 from Quantum Design. In order to study the presence of inter-cluster interactions we analyze the difference between the isothermal remanent magnetization (IRM) and the dc demagnetization (DCD) curves producing an extremely sensitive curve termed Δ*m*^24^,^29^. The values for Δ*m* are close to the noise level in our samples, thus the inter-cluster interactions can be assumed as negligible (cf. supplementary information).

Transmission Electron Microscopy (TEM) grids of Co nanoparticles covered by a carbon layer were prepared under identical conditions as the samples for magnetic experiments. We have verified in previous studies that the geometric size determined by TEM does not depend on the matrix material and corresponds closely to the magnetic size^8^,^28^. The cluster size distribution was determined from the TEM histograms adjusting a log-normal function and the results are in agreement with respect to the mean cluster size determined by TOF-MS.

**Figure 2:**
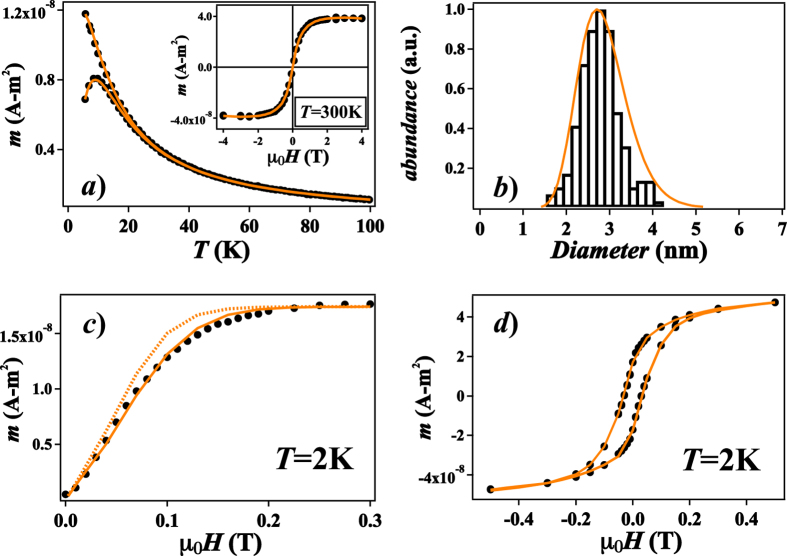
Magnetic characterization of the 2.7 nm sample. (**a**) Experimental ZFC/FC curves at 5 mT and *m*(*H*, *T*) at *T* = 300 K (points) with fits (solid lines); (**b**) comparison between geometric size distribution as derived from TEM (histogram) and log-normal magnetic size distribution as derived from triple fit; (**c**) IRM data (points) and fits with uniaxial (dashed) and biaxial anisotropy (solid line); (**d**) low temperature experimental *m*(*H*, *T*) data (points) and simulation using the parameters obtained from the fits (line).

In order to investigate the evolution of the magnetic anisotropy as a function of cluster size, a complete magnetic characterization was performed for all samples based on a series of SQUID measurements, namely zero-field-cooled/field-cooled (ZFC/FC) susceptibility, magnetization *m*(*H*, *T*) and low temperature IRM curves. The data treatment to determine the magnetic size distribution and the effective anisotropy constant *K*_*eff*_ is based on the simultaneous fit of the ZFC/FC susceptibility curves and a high temperature magnetization curve^22^. Recently this multiple fit procedure was extended to include the low temperature IRM response in order to include an anisotropy distribution described by a Gaussian function centered at 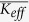
24 as well as a biaxial contribution *K*_2_ to the anisotropy^23^. Note that namely the ZFC and IRM protocols measure different magnetization reversal processes, the first being thermally activated and thus always passing via the lowest lying potential barrier between the two energy wells corresponding to a macrospin orientation along the easy axis, the second forcing the macrospin close to the field direction. Consequently the IRM measurement probes contributions beyond the uniaxial hypothesis. The obtained set of parameters is finally used to simulate the magnetization cycle at 2 K using a modified Stoner-Wohlfarth model^23^. The agreement with the experimental data is very good and the obtained values for all samples are listed in Table 1. As an example the complete set of curves, fits and simulation for the 2.7 nm sample is shown in Fig. 2, the data for the other samples can be found in the supporting information.

We find a dominant uni- or biaxial anisotropy in all our samples. The values obtained for *K*_*eff*_ are of the same order as those determined by *μ*-SQUID for individual Co clusters of comparable size and embedded in Nb and carbon matrices (75–300 kJ/m^3^)^8^,^24^,^30^. They are slightly lower than those derived for ensembles of Co clusters with mean size of 3.2 nm in Ag and Au matrices (~175 kJ/m^3^)^8^, a trend consistent with previous observations^6^.

## Discussion

We observe non-monotonous variations of a factor of two in mean effective anisotropy constant 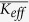 in the investigated size range as well as second-order contributions *K*_2_ to the anisotropy due to a biaxial component that are as strong as the effective uniaxial term for all but the smallest particles. Earlier measurements on cobalt nanoparticle assemblies have already investigated size effects and reported increased magnetic anisotropies with respect to the bulk value but are based on samples with strongly perturbing chemical interfaces^17^ or concentrations too high to exclude inter-particle interactions^18^. The derived values for the latter thus rather represent the barriers for switching larger domains of correlated moments in a correlated superspin glass than those of individual macrospins^31^,^32^,^33^,^34^,^35^. One way to determine the magnetic anisotropy of embedded nanoparticles is by *μ*-SQUID measurements of individual particles^30^. Albeit very elegant and powerful, this method is experimentally extremely challenging and time-consuming. Furthermore, as in all single particle measurements, it is very laborious to measure enough individual particles to obtain a statistically relevant representation of the investigated ensemble^24^.

Let us now have a closer look at the different contributions to the magnetic anisotropy^30^ and their possible size evolution. It can be written as


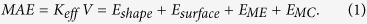


Here *E*_*shape*_ is the magnetostatic anisotropy related to the particle shape, *E*_*surface*_ is due to the symmetry breaking at the surface. In addition, if the particle experiences an external stress, the volume relaxation inside the particle induces a magnetoelastic anisotropy *E*_*ME*_. Finally *E*_*MC*_ is the cubic magnetocrystalline anisotropy energy arising from the coupling of the magnetization to the crystalline lattice as in the bulk. The MAE can be expanded in a power series of the magnetization axes in space. The shape anisotropy then only contributes with a second order term, the surface and elastic energies begin with the second order term whereas the cubic magnetocrystalline contribution starts at fourth order.

The cubic magnetocrystalline anisotropy contribution *E*_*MC*_ to *K*_*eff*_ has been determined experimentally for Co nanoparticles of 3 nm diameter to 10 kJ/m^3^, slightly lower than the bulk value for fcc cobalt of 27 kJ/m^3^^16^. Furthermore it is only for much larger cobalt nanoparticles of 20 nm diameter that in some cases a dominant cubic anisotropy was observed^36^, in our results we always find uni- or biaxial anisotropies, in agreement with *μ*-SQUID measurements of Co nanoparticles of comparable size^15^,^30^. *E*_*MC*_ is thus too small to account for our observations. The magnetoelastic anisotropy can equally be estimated negligible in our case, as the Young’s modulus of Co (209 GPa) is much bigger than for Cu (117 GPa). All strain can be expected to be relieved within the copper matrix.

We consequently retain as the two main contributions to the magnetic anisotropy of our nanoparticles the shape and the surface/interface. The shape anisotropy arises from dipolar interactions within the nanoparticle and notably induces an easy axis along the long axis of an ellipsoid. The surface magneto-crystalline anisotropy is a much more subtle contribution due to the crystalline structure and its symmetry breaking at the surface. It only becomes important in systems with a high surface/volume ratio such as thin films or small nanoparticles. If present it can, however, become the dominant term and reach extremely high values^30^,^37^,^38^.

We have characterized not only the size distribution of our deposited nanoparticles by TEM but also their shape. In order to quantify the evolution of the shape with respect to the cluster size in our experiment, we approximate the particle projections as seen in TEM by ellipses and analyze the aspect ratio *c*/*a* between the major and minor semi-axis. We adjusted a log-normal function in order to describe the aspect ratio histogram, obtaining the median value for the distribution and the dispersion *w*_*c*/*a*_ (cf. supporting information). We observe e.g. that for the sample of 2.7 nm the ratio of the two axes is close to 1, deviations from perfect spheres being at least partially due to the truncated crystalline structures and uncertainties in the image treatment which necessarily shift the mean *c*/*a* to values >1. In the case of bigger particles (>3 nm), the shape is better described by an ellipse and the median aspect ratio reaches 1.6 for the 5.5 nm clusters. We attribute this deviation from quasi-spherical shapes to the growth process in the cluster source. Prior studies have shown that large clusters produced in a laser vaporization source result from a coagulation process and are not necessarily spherical. For platinum clusters, a transition from spherical to strongly ramified shapes is observed when the cluster size increases beyond a critical diameter of about 2.5 nm^39^.

**Figure 3:**
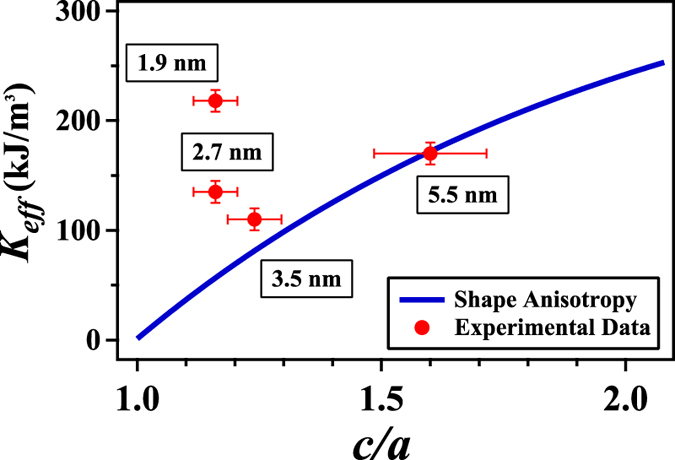
Experimental results for the mean effective anisotropy constant as a function of the aspect ratio c/a (points). The horizontal bars for the experimental data correspond to the dispersions of the aspect ratio as estimated from TEM. The blue line shows the calculated shape anisotropy constants^40^.

We calculated the demagnetizing energy for an ellipsoid from the demagnetizing factors (*N*_*xx*_, *N*_*yy*_ and *N*_*zz*_), computed by J.A. Osborn^40^ for a general ellipsoid in order to obtain the dependency of *K*_*eff*_ as a function of the aspect ratio *c*/*a* (cf. Fig. 3). Here a prolate shape corresponding to *a* = *b* < *c* implies a uniaxial anisotropy whereas a general ellipsoid with *a* < *b* < c has a biaxial component to the anisotropy. We then convert the aspect ratio distribution as derived from TEM for the two samples with the larger particles into anisotropy distributions and compare them to those derived from the fits of the magnetic data. We find reasonable agreement between the distributions for the two larger sizes within our error bars of 10% (cf. figure 9, supplementary information). Note that the derived values of *K*_2_ for the largest clusters correspond to an aspect ratio *c*/*a* of ~1.3. We can thus conclude that for elongated nanoparticles the shape anisotropy is very important and can account by itself for the comparably high values derived in our experiment. This finding, however, cannot explain the increase by a factor of two when decreasing the size from 3.5 nm to 1.9 nm. For example a too small mean value of *K*_*eff*_ ~ 60 kJ/m^3^ due to the shape is calculated for the aspect ratio *c*/*a* = 1.16 determined for the 2.7 nm sample.

In the size regime below 3 nm the fraction of atoms at the surface of a quasi-spherical particle is above 30%, the role of the surface becomes more and more important. We will consequently now try to correlate the observed rise in anisotropy to the increased importance of the surface. An established model to calculate surface contributions to the magnetic anisotropy is the Néel pair model^41^ which has successfully been used for both thin films as well as for nanoparticles^21^,^30^,^42^,^43^,^44^. The close agreement between a more elaborate quantum mechanical approach and the Néel model calculations for thin films further supports this phenomenological approach^45^. In this model the pair interaction is described by one single parameter derived from magneto-elastic constants, therefore intimately connected to lattice variations due to relaxation as well as to symmetry breaking at the cluster surface. Notably the main contributions arise from additional facets to the cluster, a closed shell structure does not result in a specific surface anisotropy^42^. For the following calculations we assume closed shell truncated fcc octahedra with one additional facet for the geometric structure and use a value of *L* = −15 MJ/m^3^ for the Néel constant^30^. Note that the closed shell structures only display cubic magnetocrystalline anisotropy of the order of 10 kJ/m^3^^30^ and that the addition of atoms at random positions of the particle surface only results in a small increase of anisotropy (~50 kJ/m^3^)^42^, insufficient to explain our experimental findings. Adding a single facet does not significantly modify the shape of the particle but it breaks the symmetry and thereby induces an additional and very large anisotropy for the entire particle. A single unsupported facet of one atomic layer thickness will not have the same magnetic anisotropy. The resulting cluster keeps an aspect ratio close to 1, we consequently neglect shape contributions to the anisotropy in this part of the discussion.

**Figure 4:**
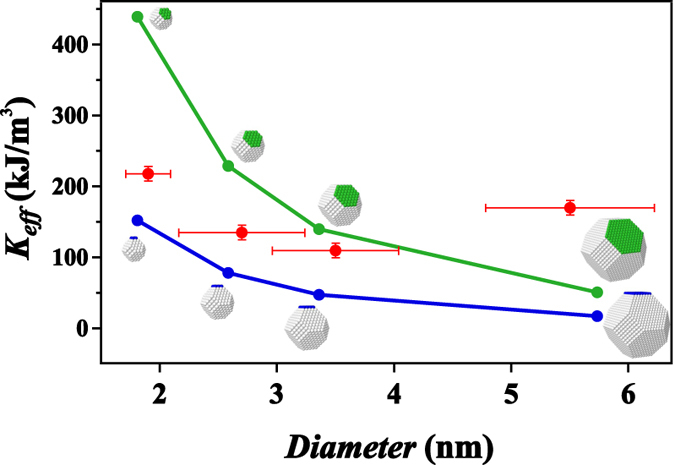
Comparison of the experimental values for 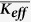 with the magnetic surface anisotropy calculated using the Néel model for different example cluster sizes and single facets added along the [100] (blue, lower curve) and [111] (green, upper curve) directions. The horizontal bars for the experimental data correspond to the dispersions obtained for magnetic cluster size from the fits. The simulated structures close to 6 nm are only included to show the general trend of the surface anisotropy and have no relation to the particle shapes observed in the experiment. The aspect ratio for all calculated structures is close to one.

In order to test whether the surface anisotropy contribution can be responsible for the increased measured values for small sizes, we calculate its magnitude for a number of suitable geometric structures of truncated fcc octahedra with additional facets. Fig. 4 shows values for octahedra of different size with one additional facet added along either the 100 or the 111 direction. Clearly it is possible to reproduce both observed effects, the increased values for the anisotropy constant with respect to the bulk for small sizes as well as its trend with decreasing size. Here we only consider a small number of highly symmetric structures, the size dispersions of the experimentally investigated clusters naturally imply a very large number of different partially or completely filled additional facets. This dispersion leads to very large variations of the calculated surface anisotropy, ranging from zero for a filled geometric shell to values even bigger than the experimental ones for highly anisotropic structures. We consequently do not claim the displayed geometric structures depicted in Fig. 4 to be the only correct ones but take them as examples for the increasing role of the nanoparticle surface at small sizes. We do not suppose all our clusters to be crystallized in a fcc structure. Several studies of metal clusters of different materials and for different sizes have observed the coexistence of various crystallographic structures in both the gas as in the deposited phase^46^,^47^,^48^,^49^, especially without further annealing. Comparable strong variations with values of the same order (between 10 and 400 kJ/m^3^) have also been derived for icosahedral structures in the size regime between 3.1 and 4.3 nm^21^. As hexagonal structures in Co nanoparticles are only observed at sizes above 20 nm^50^ we can consequently expect crystalline structures based on fcc to be dominant and the general trend described in this article to be independent of the exact crystalline structure of the nanoparticles in question.

Note that the addition of several facets along different crystalline axes does not lead to a summed anisotropy constant but rather to higher order corrections beyond uniaxial anisotropy. We find large values of *K*_2_ for the bigger clusters in the experiment and explain them as due to the non-sphericity of the nanoparticles. In the intermediate size range *K*_2_/*K*_*eff*_ is determined by both shape and surface effects and our values are in good agreement with previously obtained data from *μ*-SQUID measurements^15^,^42^.

Finally we would like to underline the complexity of the problem. Even though our results show that the increase in magnetic anisotropy due to the addition of a single facet decreases for larger particles (although it contains more atoms), we find that it is nevertheless possible to obtain very large values for *K*_*eff*_ by adding several facets. For a particle of approximately 6 nm diameter with a resulting aspect ratio close to the experimentally observed value we calculate for specific crystalline structures values of the order of what we obtained from shape anisotropy. This means that notably polycrystallinity, which we can expect in large particles due to the fabrication mechanism, plays an important role in reducing the contributions of crystal-symmetry breaking to the global anisotropy. Defects have, on the other hand, been proposed as being responsible for uniaxial anisotropy in larger cobalt or iron nanoparticles (8–20 nm)^36^,^51^. The detailed study of the crystalline structures of the nanoparticles and their exact relation with the magnetic anisotropy as calculated by the Néel model is, however, beyond the scope of this article and will be addressed in the future. The work presented here illustrates the crucial role of the surface for small nano-magnets, and its possibility to induce a size-dependent magnetic anisotropy.

## Conclusions

We have demonstrated the extraction of reliable magnetic parameters of embedded cobalt clusters and evidenced a non-monotonous variation of a factor of two of the magnetic anisotropy with cluster size. The values for clusters with diameters >3 nm can in our case be reproduced by simply converting deviations from a spherical shape into shape anisotropy. For smaller particles the surface has to be taken into account and we show that both the magnitude of the experimentally derived values as well as the increase with decreasing size can be reproduced with simulations based on the Néel pair interaction model. These observations underline the importance of the shape and the addition of facets to the nanoparticle. Our experiments are to be seen complementary to previous work on individual particles and open the way to the rapid and accurate characterization of cluster-assembled nanostructures. Furthermore they provide crucial input for continuing theoretical investigations of the magnetic anisotropy in nanoscale systems on a true quantum mechanical level. Notably the effect of the interface on spin-orbit coupling needs to be taken into account in ab-initio calculations as it has been proven essential in thin film systems.

## Additional Information

**How to cite this article**: Oyarzún, S. *et al.* Size effects in the magnetic anisotropy of embedded cobalt nanoparticles: from shape to surface. *Sci. Rep.* **5**, 14749; doi: 10.1038/srep14749 (2015).

## Supplementary Material

Supplementary Information

## Figures and Tables

**Table 1:** Parameters obtained by the fits of the magnetic data for samples with different cluster size.

Sample	*D*_*mag*_ (nm)	*w*_*mag*_	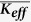 (kJ/m^3^)	*w*_*K*_	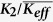
1.9 nm	1.9	0.10	218	0.40	0
2.7 nm	2.7	0.20	135	0.40	0.8
3.5 nm	3.2	0.17	110	0.35	1.1
5.5 nm	5.5	0.13	170	0.35	1.2

The errors for the values are 0.1 nm for the median magnetic diameter *D*_*mag*_, 0.01 for the diameter dispersion *w*_*mag*_ in a log-normal description, 10 kJ/m^3^ for the effective anisotropy constant *K*_*eff*_, 0.1 for the Gaussian anisotropy dispersion *w*_*K*_ and 0.4 for the anisotropy ratio *K*_2_/*K*_*eff*_. The error values were estimated in a thorough study of the confidence limits of the fit procedures^22^,^23^,^24^.
