# Supplementary material for: Size effects in the magnetic anisotropy of embedded cobalt nanoparticles: from shape to surface
Source: Sci Rep. 2015 Oct 6;5:14749. doi: 10.1038/srep14749 (PMC4593963; doi:10.1038/srep14749)
Supplement: Supplementary Information [file srep14749-s1.pdf]

# Size effects in the magnetic anisotropy of embedded cobalt nanoparticles: from shape to surface.

Simon Oyarzun, Alexandre Tamion, Florent Tournus, Véronique Dupuis, and Matthias Hillenkamp

Institut Lumière Matière, UMR5306 Université Lyon 1-CNRS, Université de Lyon, 69622 Villeurbanne cedex, France

corresponding author: matthias.hillenkamp@univ-lyon1.fr

# Supplementary information

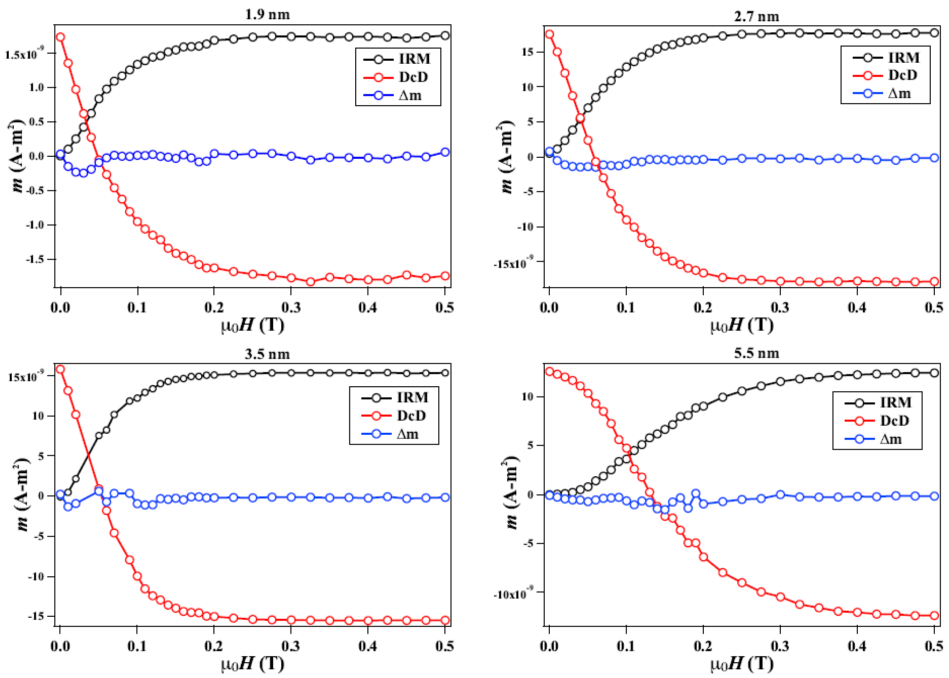


Figure 5: Isothermal Remanent Magnetization (IRM) and Dc Demagnetization (DCD) curves for the four samples as well as the weighted difference:

$$\Delta m = DCD(H) - \left(m_R - 2IRM(H)\right),$$

where $m_R$ is the remanent magnetization after saturation. Significant deviations from $\Delta m = 0$ are indications for inter-particle interactions. The noise level in our experiments is about $1*10^{-10}$ Am² ($10^{-7}$ emu). For further details on the technique see references [1, 2].

Figure 6a: 1.9 nm diameter

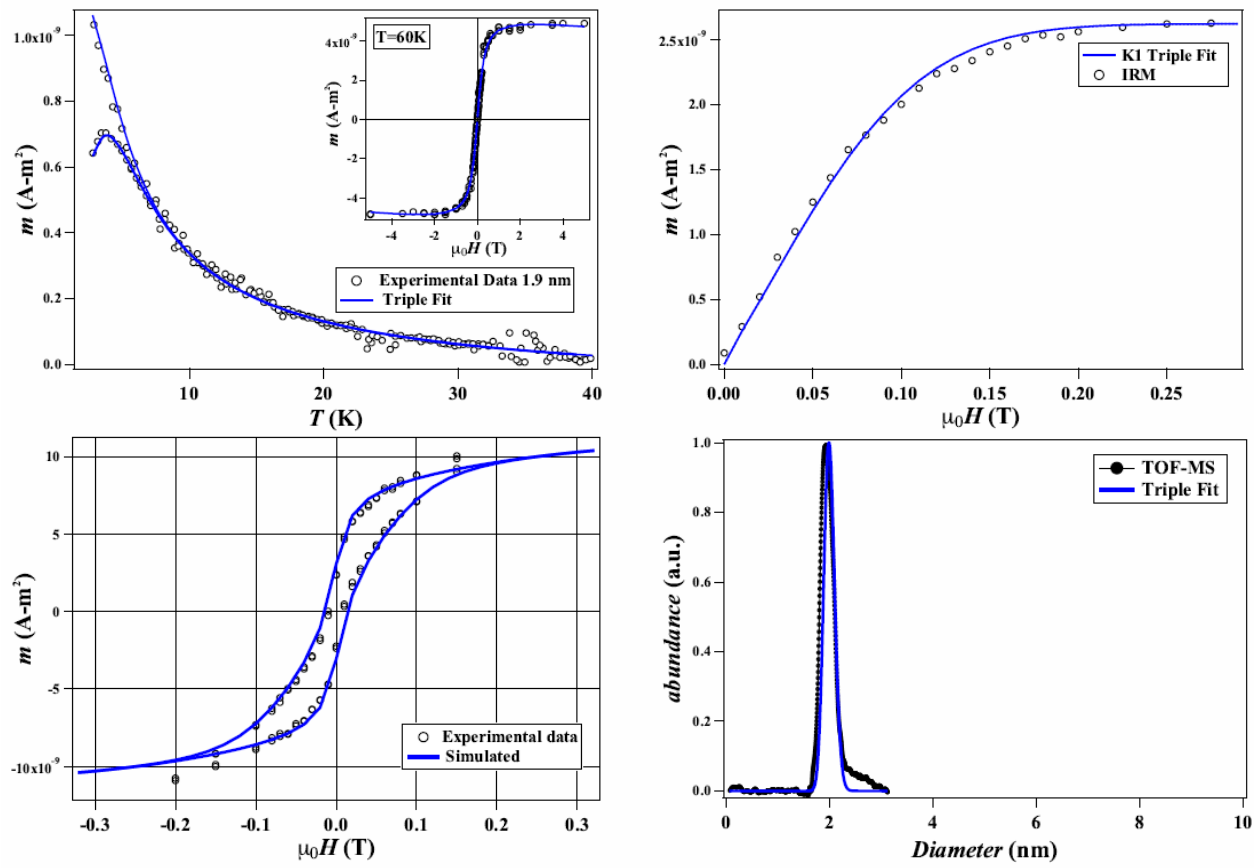


Figure 6b: 2.7 nm diameter

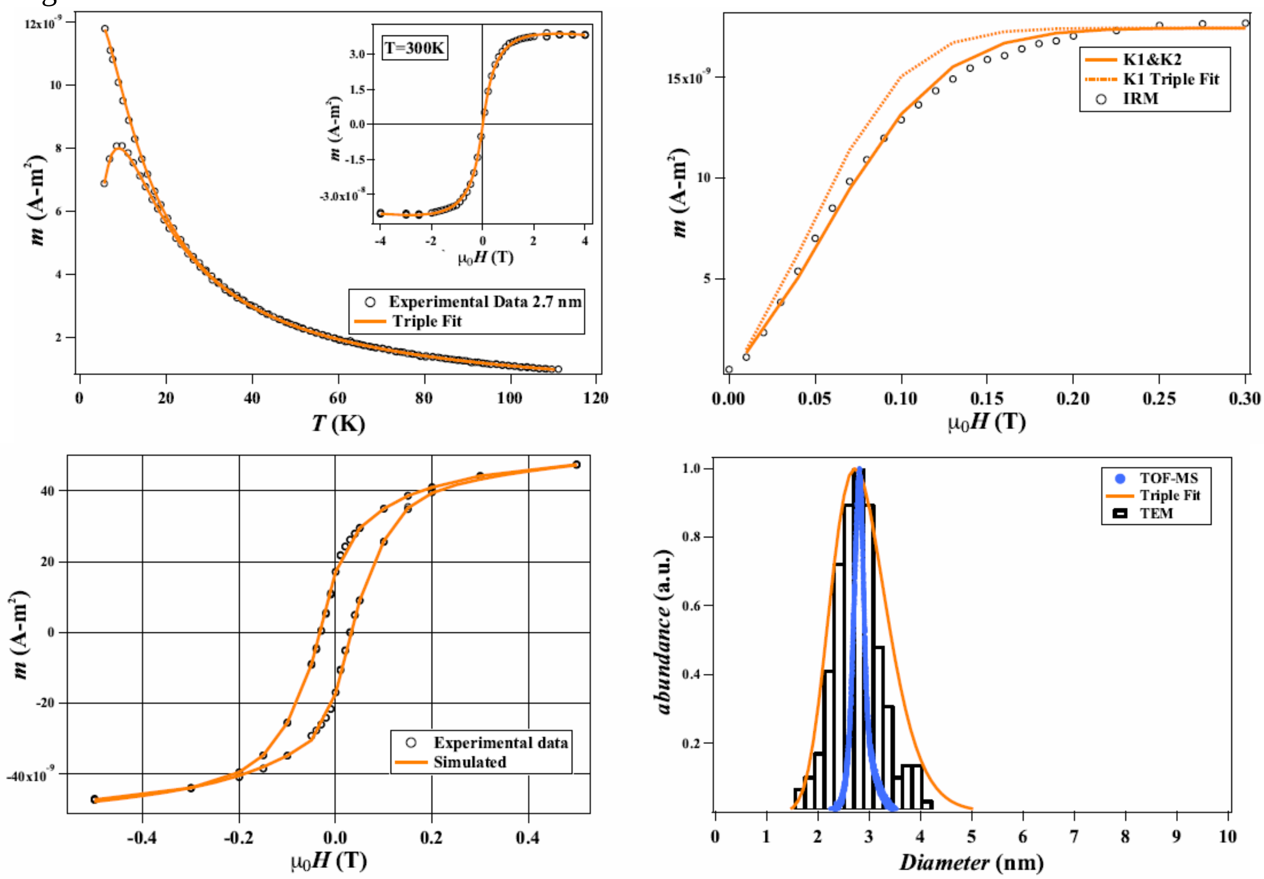

Figure 6c: 3.5 nm diameter

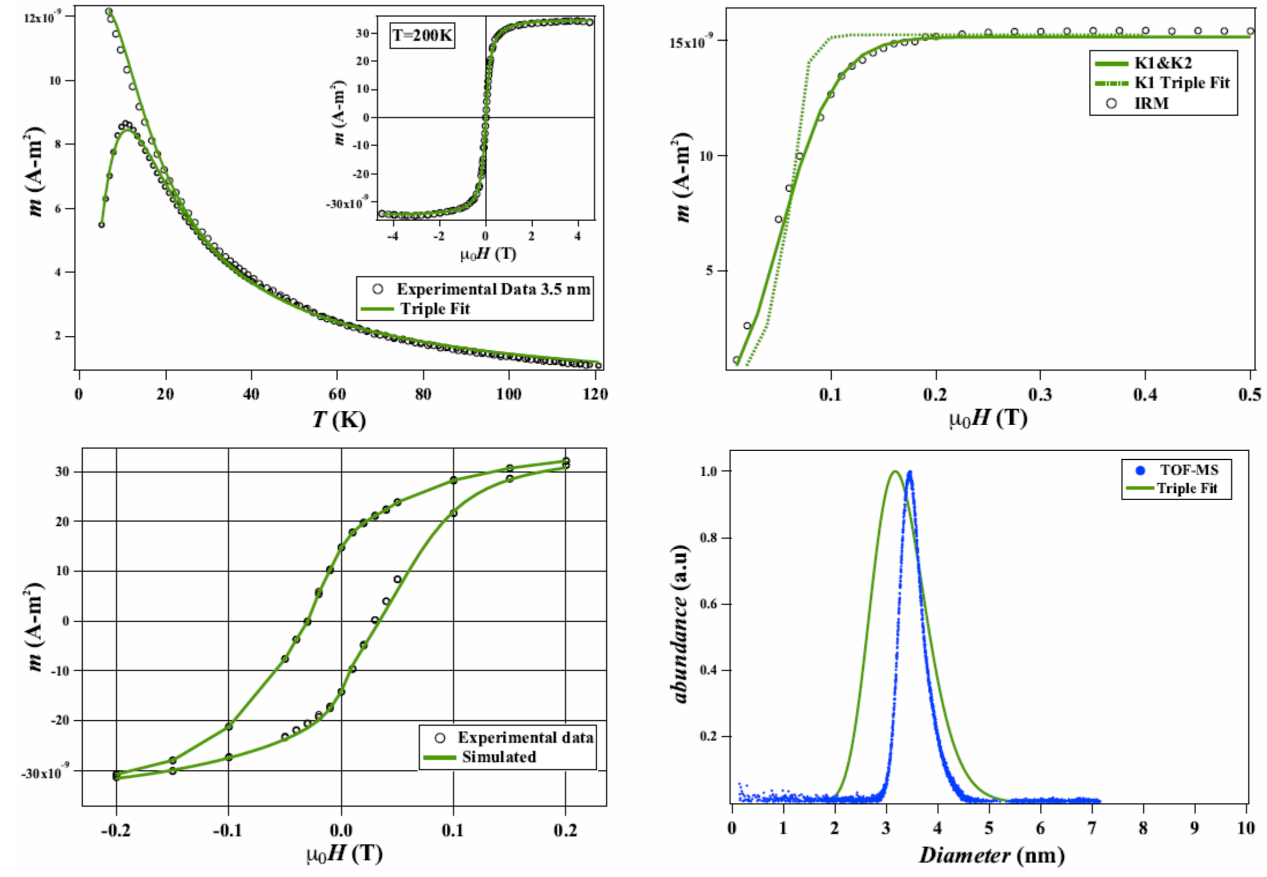


Figure 6d: 5.5 nm diameter

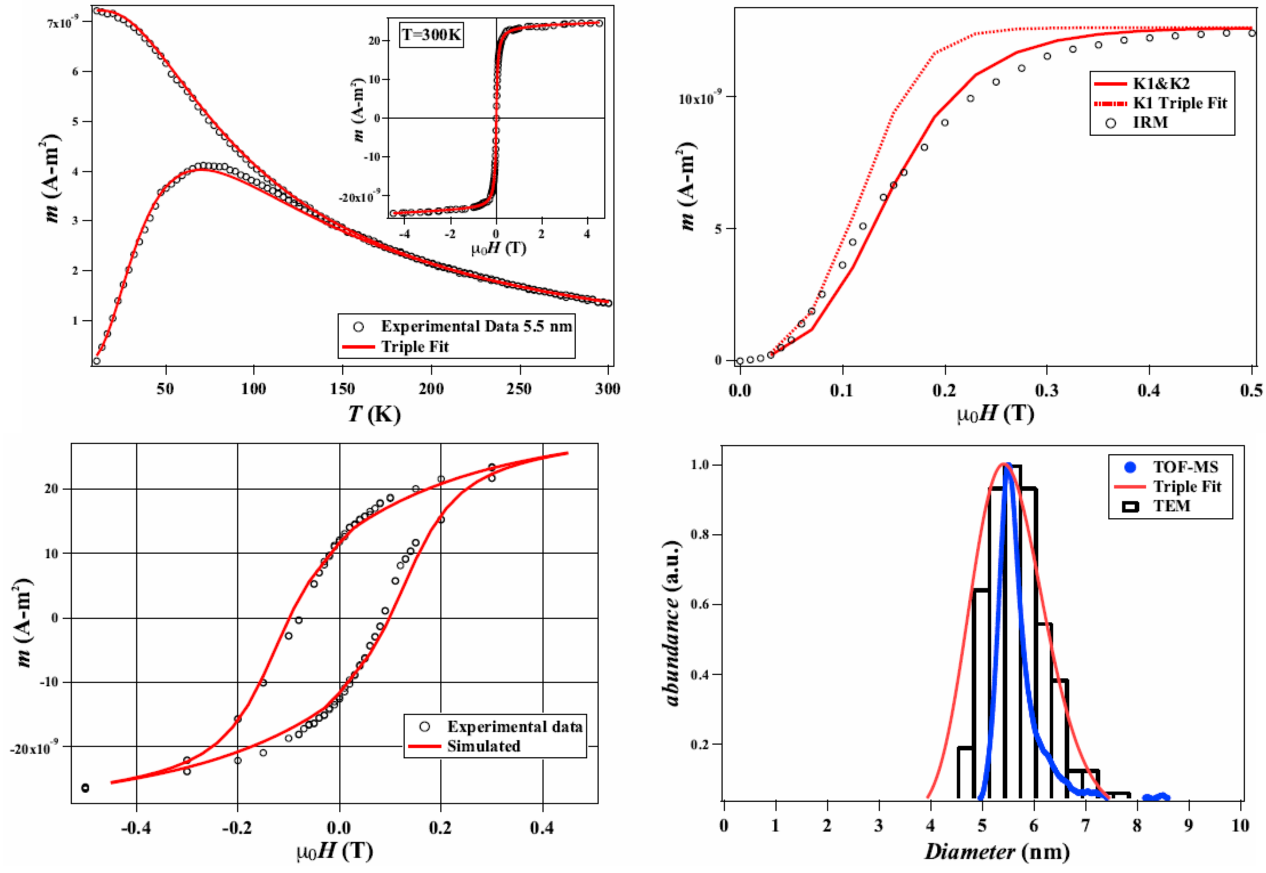

Figure 6: Experimental data and fits for all four samples investigated. The color codes correspond to the mass spectra of figure 1 in the text (blue: 1.9 nm diameter; yellow: 2.7 nm; green: 3.5 nm; red: 5.5 nm).

left top) Experimental ZFC/FC curves at 5 mT and $m(H,T)$ at $T \gg T_{max}$ (points) with fits (solid lines);

right top) comparison between size distributions as derived from MS-TOF, TEM and magnetic fits;

left bottom) IRM data (points) and fits with uniaxial (dashed) and biaxial anisotropy (solid line);

right bottom) low temperature experimental $m(H,T)$ data (points) and simulation using the parameters obtained from the fits (line).

The low temperature magnetization cycles of the two smallest samples are superpositions of a blocked and a superparamagnetic fraction of the size distribution, leading to “wasp tail” curves. Only for the smallest clusters a satisfactory fit of the IRM curves could be obtained without biaxial anisotropy term, for the other samples a fit with only uniaxial anisotropy was not sufficiently good.

The characterization of the clusters in the gas phase by time-of-flight mass spectrometry is a rapid indication of the mean size indispensable for tuning and verification throughout the sample fabrication. The final size dispersion of deposited particles is, however, underestimated, most probably due to ion optics effects. The more reliable comparison is the one between deposited clusters, as has been verified in several previous studies [3, 4, 5, 6].

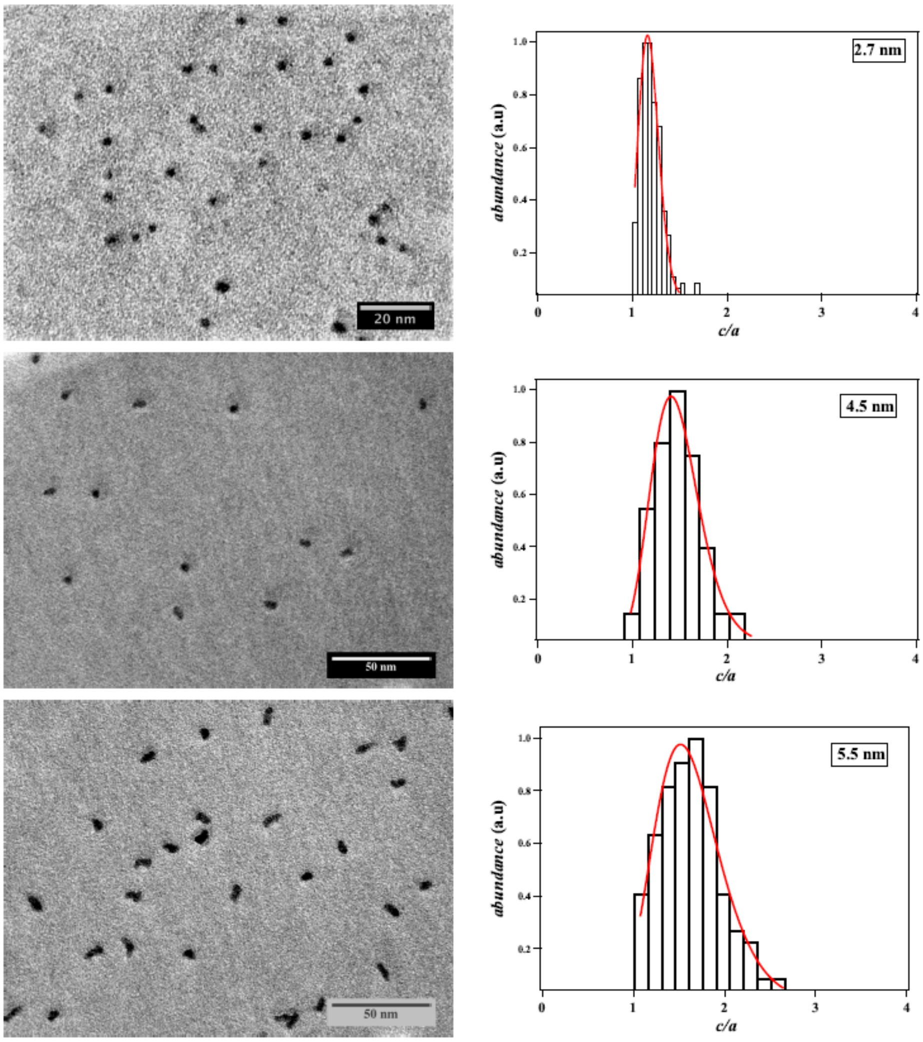


Figure 7: Transmission Electron Micrographs for three different cluster sizes and the corresponding aspect ratio distributions. The distributions are well reproduced by truncated log-normal functions.

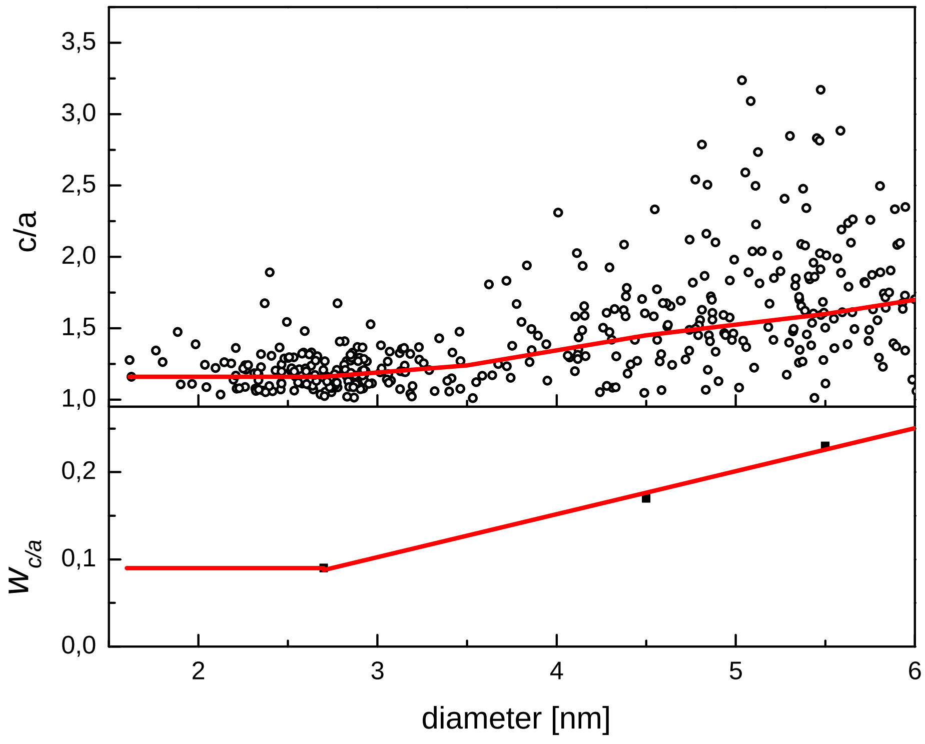


Figure 8: Evolution of the aspect ratio and the dispersion as derived from fitting an ellipse to the cluster projections in TEM. The increase of both the mean *c/a* as well as its dispersion is clearly visible. For sizes below 2.7 nm we assume a constant ratio *c/a* of 1.16 and $w_{c/a}$ = 0.09. The values according to the truncated log-normal distribution for 3.5 nm were obtained by interpolation.

The c/a values have uncertainties notably due to the discrete pixel structure of the images and possible aberrations which can effectively shift the mean c/a to slightly bigger values for the smallest particles. We have estimated the uncertainty in the image analysis due to a pixel size of ~0.1 nm, resulting for hypothetical spherical clusters of 2 nm diameter in an uncertainty for *c/a* of 10% corresponding to a shift up to *c/a* = 1.1. Note that in our samples the dispersion is much bigger and dominates the uncertainties.

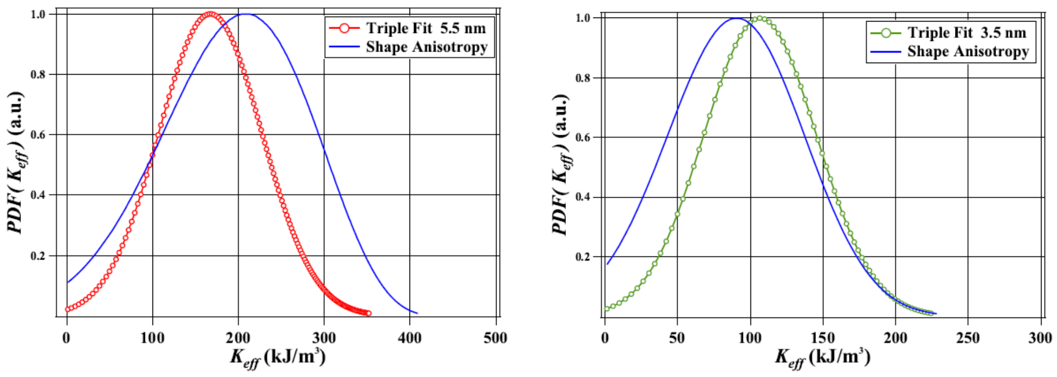


Figure 9: Anisotropy dispersions as derived from the triple fits of the magnetic data (dots) and from the conversion of the aspect ratio distributions into shape anisotropy (full lines).

References


[1] Hillion, A., Tamion, A., Tournus, F., Gaier, O., Bonet, E., Albin, C., and Dupuis, V. *Phys. Rev. B* **88**, 094419 (2013).

[2] Kelly, P., O'Grady, K., Mayo, P., and Chantrell, R. W. *IEEE Trans. Magn.* **25**, 3881 (1989).

[3] Tamion, A., Hillenkamp, M., Tournus, F., Bonet, E., and Dupuis, V. *Appl. Phys. Lett.* **95**, 062503 (2009).

[4] Tamion, A., Raufast, C., Hillenkamp, M., Bonet, E., Jouanguy, J., Canut, B., Bernstein, E., Boisron, O., Wernsdorfer, W., and Dupuis, V. *Phys. Rev. B* **81**, 144403 (2010).

[5] Tamion, A., Hillenkamp, M., Hillion, A., Tournus, F., Tuaillon-Combes, J., Boisron, O., Zafeiratos, S., and Dupuis, V. *J. Appl. Phys.* **110**, 063904 (2011).

[6] Oyarzún, S., Domingues Tavares de Sa, A., Tuaillon-Combes, J., Tamion, A., Hillion, A., Boisron, O., Mosset, A., Pellarin, M., Dupuis, V., and Hillenkamp, M. *J. Nanopart. Res.* **15**, 1968 (2013).
